# Acceptability of donated breast milk among pregnant women in selected hospitals in central Uganda: a cross-sectional study

**DOI:** 10.1186/s13006-023-00569-x

**Published:** 2023-06-16

**Authors:** Mary Gorreth Namuddu, Juliet Kiguli, Victoria Nakibuuka, Ritah Nantale, David Mukunya

**Affiliations:** 1 School of Public Health, College of Health Sciences, Makerere University, Kampala, P.O. Box 7072, Uganda; 2 Department of Paediatrics, Nsambya Hospital, Kampala, P.O. Box 7146, Uganda; 3 Department of Community and Public Health, Faculty of Health Sciences, Busitema University, Mbale, P.O. Box 1460, Uganda; 4 Department of Research, Nikao Medical Center, Kampala, Uganda

**Keywords:** Human milk banking, Acceptability, Donated breast milk, Breastfeeding.

## Abstract

**Background:**

Donated breast milk is considered beneficial to vulnerable infants. Thus, Uganda launched its first human milk bank in November 2021 to provide breast milk to preterm, low birthweight and sick babies. However, there is a scarcity of information on the acceptability of donated breast milk in Uganda. The study sought to assess the acceptability of using donated breast milk and associated factors among pregnant women at a private and a public hospital in central Uganda.

**Methods:**

This cross-sectional study enrolled pregnant women attending antenatal care at the selected hospitals between July and October 2020. All pregnant women recruited had already given birth to at least one child. Data were collected using a semi-structured questionnaire, and we recruited participants through systematic sampling. Used frequencies, percentages and means with standard deviations to summarize variables. Assessed the association between the acceptability of donated milk and selected factors by comparing their arithmetic means using a generalized linear model to allow for clustering at the health facility level. Used a normal distribution and an identity link and calculated the adjusted mean differences together with 95% CIs [generated using robust variance estimators to correct for model misspecification].

**Results:**

A total of 244 pregnant women with a mean age of 30 (± 5.25) years were enrolled. Sixty-one-point 5% (150/244) of the women reported that they would accept donated breast milk. Higher education (adjusted mean difference, technical versus primary level: 1.33; 95% CI 0.64, 2.02), being Muslim (adjusted mean difference, Muslim versus Christian: 1.24; 95% CI 0.77, 1.70), having heard of donated breast milk banking (adjusted mean difference, ever versus never: 0.62; 95% CI 0.18, 1.06) and presence of a serious medical condition (adjusted mean difference, preference of donated milk versus other feeds in a serious medical condition: 3.96; 95% CI, 3.28, 4.64) were associated with acceptability of donated breast milk.

**Conclusions:**

The acceptability of using donated breast milk for infant feeding was high among pregnant women. Public sensitization and education campaigns are indispensable for the acceptability of donated milk. These programs should be designed to include women with lower education levels.

**Supplementary Information:**

The online version contains supplementary material available at 10.1186/s13006-023-00569-x.

## Background

Evidence is clear that breast milk protects infant health. Worldwide, optimal breastfeeding alone could prevent over 800,000 child deaths [1]. However, maternal challenges (delayed milk production, birth trauma, abandonment, maternal stress and illnesses) and infant challenges (illness, inability to suckle) render some mothers unable to produce enough breast milk for their babies. In such cases, donated breast milk is recommended as the first option for supplementation [2, 3]. Donated breast milk is milk expressed by a mother, processed by a human milk bank and then given to a child that belongs to another mother [4]. Donated breast milk benefits vulnerable infants, especially premature and low birthweight babies [5]. It is associated with a reduced risk of necrotizing enterocolitis, diarrhea, feeding intolerance, improved cognitive development, retinopathy, late-onset sepsis and other infections [2, 6, 7]. Compared to infant formula, donated breast milk is cost-effective because babies tend to tolerate full feeds rapidly when fed donated milk and stay hospitalized for a shorter time [8, 9]. In addition, donated breast milk increases awareness of the value of breastfeeding and encourages mothers to breastfeed exclusively, especially when there is support for optimal lactation [4, 10, 11].

Despite the benefits, studies worldwide show contradicting findings regarding accepting donated breast milk for infant feeding. According to Muslim scholars, human breast milk creates milk kinship [12]. Hence, breast milk shared in any mode may not be used among Muslims [12]. In Africa, findings are different across regions. For instance, Kimani-Murage et al. (2019) found that at the individual level, two-thirds of the participants in Kenya were willing to accept feeding their babies on donated breast milk [13]. However, a mixed-methods study in eastern Ethiopia conducted among mothers revealed that only 15% of respondents were willing to use donated breast milk [14]. Similarly, Abhulimhen-Iyoha et al. (2015) reported in their study that 84% of the mothers in Nigeria would not give their babies donated breast milk [15]. Unfortunately, low-income countries still lag in implementing human milk banking [4, 16]. When mothers do not have breast milk, they opt for prelacteal feeds and subsequently mixed feeding, predisposing the newborns to neonatal infections, diarrhea, and increased risk of death. In Uganda, over 34% of newborns do not initiate breastfeeding within the first hour, and neonatal deaths account for 42% of deaths among children below five years [17, 18].

In response to the need for a life-saving alternative for vulnerable infants, Uganda launched its first human milk bank at St. Francis Hospital Nsambya in November 2021 [19]. The bank intends to provide donated breast milk to preterm infants, low birthweight, and sick babies that cannot access their mother’s breast milk. However, little is known about the acceptability and factors associated with the use of donated breast milk for infant feeding in Uganda. Several factors could influence the acceptability of donated breast milk use. For instance: maternal education, cultural and religious beliefs, knowledge of donated milk and the value of breast milk, experience in wet nursing, antenatal visits, having a baby in the neonatal intensive care unit, safety issues, family/health workers’ support, and breastfeeding/birth complications [4, 13, 14, 20–24]. The differences in the level of acceptability, even within the same regions, call for a need to generate contextual data. In this study, acceptability is “a multi-faceted construct that reflects the extent to which people receiving a healthcare intervention consider it to be appropriate, based on anticipated or experienced cognitive and emotional responses to the intervention” [25]. Ascertaining the acceptability of donated breast milk and associated factors among would-be users will help provide adequate information to address misconceptions and beliefs regarding the milk. Hence, implement the bank successfully and increase acceptability. This study sought to assess the acceptability of the use of donated breast milk and associated factors among pregnant women at Nsambya and Naguru hospitals in Kampala, Uganda.

## Methods

### Study design and setting

We conducted a hospital-based cross-sectional study at a private hospital (St. Francis Hospital Nsambya) and a public hospital (Naguru/China-Uganda Friendship Hospital) in Kampala district, the capital city of Uganda. Hospitals were selected using purposive sampling. Nsambya Hospital is a tertiary referral hospital with approximately 35 to 40 premature infants registered each month [26, 27]. The hospital is known for its expertise in caring for premature babies. The first human milk bank in Uganda was established at this hospital. In collaboration with the Ministry of Health, the hospital is leading the implementation of donated breast milk use in Uganda to ensure that vulnerable infants such as the sick, premature, and low birthweight receive the best possible nutrition. Naguru Hospital; is one of the referral public hospitals designed to decongest Mulago national referral hospital [28]. The hospital receives patients from all over the country and registers many preterm births [29]. Also, it is close to Nsambya Hospital, thus easy to access. The two hospitals selected are involved in caring for preterm infants, the main beneficiaries of donated breast milk. Also, carrying out the study in private and public hospitals enabled determining the acceptance of donated breast milk from different categories of potential users.

### Study population

The study involved pregnant women at any stage of pregnancy who had had at least one child and attended the antenatal unit at the selected hospitals from July to October 2020. We only included participants who gave informed written consent, treated women below 18 years as emancipated minors and excluded women who were unwell. According to the Uganda National Council for Science and Technology, emancipated minors are individuals below 18 years who are either pregnant, married, have a child or are self-sufficient. In Uganda, emancipated minors can independently consent to participate in research if approved by a research ethics committee (REC) [30].

### Sample size

The sample size was determined using Fleiss’s formula for comparing two independent proportions [31]. We assumed a 95% confidence level, a power of 80% and a ratio of 2:1 (exposed to unexposed). To determine the sample size needed to detect factors associated with the acceptability of donated breast milk. We calculated sample sizes of various exposures reported in the literature to be significantly associated with acceptance of the use of donated breast milk. These included; ever heard about human milk banking, ever visited the neonatal intensive care unit (NICU), ever heard about wet nursing. Computations were done using StatCalc for EpiInfo (version 7.2.3.1). Compared to other factors, visiting NICU gave the largest sample size, hence considered. We assumed a 31.48% proportion of acceptability among those exposed to NICU and a 14.31% proportion of acceptability among the unexposed to NICU, as reported by Gelano et al. (2018) [14]. Yielding a total sample size of 216 and adjusting for a non-response of 10% gave a minimum sample size of 240 respondents for the study. Using proportional-to-size, we distributed the sample size in each hospital based on the monthly number of pregnant women attending the antenatal units. Therefore, 141 respondents were to be recruited from Nsambya Hospital and 99 from Naguru Hospital. To obtain the sample needed per study day, we considered the sample size allocated as a proportion of the total number of days for data collection from each hospital. Twenty-four and 25 eligible pregnant women were enrolled per study day at Nsambya and Naguru hospitals, respectively. In the end, we recruited 144 respondents from Nsambya Hospital and 100 respondents from Naguru Hospital, resulting in a total of 244 respondents as the final sample size for the study.

### Sampling procedure

Respondents were enrolled using systematic sampling based on the calculated interval of three. We determined the sampling interval by considering the number of pregnant women who come for antenatal visits in a day in each hospital from the registration log books and the sample needed per study day. On average, Nsambya Hospital had 80 pregnant women, and Naguru Hospital had 70 pregnant women visiting the ANC units. We used these numbers as the sampling frame for all the study days. All eligible respondents were given a card with a number as they came in. Then we randomly sampled from the first three respondents and picked the second as a starting point. From the second respondent, we consistently selected every 3rd respondent until we obtained the number needed.

### Data collection and management

Data collection started with obtaining consent from the respondents. Respondents were presumed to be relatively unaware of human milk banking, so we briefly explained what it is and the concept of donated breast milk. Two research assistants and the principal researcher collected the data. Data were collected using an interviewer-administered semi-structured questionnaire. The questionnaire was programmed in Open Data Kit (ODK) collect electronic platform and uploaded on Android tablets for data collection. The questionnaire was prepared in English and translated into Luganda, the most spoken local dialect. The questionnaire was pre-tested on 5% of the final sample size and comprised of questions that assessed the acceptability of donated breast milk and the associated factors. The research assistants were trained and involved in pre-testing the tool before data collection. The training focused on the study objectives, the content of the questionnaire and how to conduct interviews. The data collected was checked daily for completeness, identified and corrected errors, and subsequently submitted to the Ona server and then exported to Ms Excel. The following are the study variables collected.

### Outcome variable

*Acceptability of the use of donated breast milk* as a dependent variable was measured using the Theoretical Framework of Acceptability (TFA) recommended by Sekhon et al., 2017 [25]. TFA was selected because it can assess the acceptability of healthcare interventions of either service providers or recipients before delivery. The study used a questionnaire with 1 question per TFA construct to measure acceptability. Respondents had to rate the TFA measures on a 5-point Likert scale (1- strongly disagree to 5- strongly agree) [25]. A higher score reflected high acceptability. Negatively stated variables were reverse coded. Then we generated a continuous variable with a minimum possible score of 6 and a maximum possible score of 30; and treated the scale as a whole. A total score for the six constructs reflected respondents’ acceptability. The scale had a good level of internal consistency (Cronbach’s alpha 0.71).

### Independent variables

Andersen and Newman’s model of health service utilization (fourth phase)[32, 33] with prior research was employed to understand factors associated with the acceptability of donated breast milk. This study specifically focused on individual determinants of health service use. The model assumes that individuals use health services depending on predisposing, enabling and need factors. Predisposing factors refer to personal characteristics such as demographics, health beliefs, and social structure that influence the use of health services even before the onset of a specific illness. Enabling factors are related to the availability of health service resources and may originate from community or family support. While need factors are related to the individual’s health condition and are among the most immediate cause of accepting health services. Table 1 shows the definitions for the constructs used to measure acceptability as described by Sekhon et al., and other measures for the study variables.

**Table 1:** Summary of definitions and measures for the study variables

Outcome variable; Acceptability of the use of donated breast milk		
***TFA Constructs***	***Definition***	***Measures***
Anticipated affective attitude	How an individual feels about the intervention before taking part.	I believe donated breast milk from a milk bank provides the best alternative source of food for babies in the absence of the biological mother’s milk.
Ethicality	The extent to which the intervention has a good fit with an individual’s value system.	Feeding babies donated breast milk is ethically acceptable (It is not against my values and beliefs).
Anticipated effectiveness	The extent to which the intervention is perceived to be likely to achieve its purpose.	I believe feeding babies donated breast milk from a milk bank prevents childhood illness and has health benefits comparable to the biological mother’s milk.
Anticipated burden	The perceived amount of effort required to participate in the intervention	Using donated breast milk from a milk bank is a complex process and requires a lot of time or expense.
Anticipated opportunity cost	The extent to which benefits, profits, or values must be given up to engage in the intervention	In my opinion, I would be willing to give up the convenience of the use of infant formula or cow milk for donated breast milk.
Self-Efficacy	Participant’s confidence that they can perform the behaviour required to participate in the intervention	If I didn’t have enough breast milk or was unable to breastfeed, I would feed my baby on donated breast milk.
Independent variables; predisposing, enabling and need factors.		
*Measures*		
*Sociodemographic characteristics*		
Age		15–24; 25–44; ≥45 years.
Religion		Christianity; Islam
Marital status		Single; married/cohabiting; divorced/separated; widowed
Education level		None; Primary; Secondary; Vocational/technical; University
Occupation		Housewife; self-employed; salaried/waged
Mode of delivery of the current child		Cesarean section; normal delivery
Parity		1–3; ≥4 children
Income		Self-reported based on the average monthly family income
Number of antenatal visits (ANC)		1–3; ≥4 times
Ever heard about donated breast milk		Yes; no
Ever heard about donated breast milk banking		Yes; no
Ever heard about wet nursing		Yes; no
Ever breastfed another person’s child		Yes; no
Ever been counselled on breastfeeding during ANC visits		Yes; no
Require consent from a spouse		Yes; no
Preferred feed in presence of a serious medical condition		Donated breast milk; other feeds
Ever visited/ admitted to the neonatal intensive care unit (NICU)		Ever; never
Donor type		Relative; friend; human milk bank; none

### Data analysis

We summarized continuous variables using means with standard deviations and categorical variables using their frequencies and percentages to describe the characteristics of the respondents and the data obtained. To determine the level of acceptability, we categorized the data to differentiate acceptability and non-acceptability. A cut-off point of 95%CI of the mean of the total score was used, as reported in prior research [34]. Women who scored above two standard deviations of the mean were considered to have accepted the use of donated breast milk, and those who scored below two standard deviations of the mean not to have accepted.

We checked for assumptions, and the probability plots (Additional Fig. 1) and Shapiro-Wilk test (*p* - value of 0.79) suggested that the residuals were approximately normally distributed. The scatterplot of “residuals versus fitted values” (Additional Fig. 2) indicated that the residual distribution was narrowing towards the right. Also, the Breusch-Pagan test (*p* - value < 0.001) showed no constant error variance. The mean VIF value obtained was 1.35, and the tolerance (1/VIF) was high, with all the explanatory variables exceeding 0.2. Hence no multicollinearity existed within the data. The cook’s distance test based on the threshold of 1 showed that none of the cases was influential. All the values obtained were less than 1, with a mean of 0.008 ± 0.048.

To explore the association between the acceptability of donated milk and selected factors, we compared their arithmetic means using a generalized linear model to allow for clustering at the health facility level. A normal distribution and an identity link were used, and the adjusted mean differences were calculated together with 95% CIs [generated using robust variance estimators to correct for model misspecification]. We analyzed acceptability as a continuous outcome based on the respondent’s total score on the six constructs. Based on scientific literature, the following explanatory variables were added to the model; Age group, mothers’ education level, occupation status, mother’s religion, number of times a mother visited the ANC, ever been counselled on breastfeeding at ANC, ever visited the NICU, ever heard about Donated Breast Milk Banking (DBMB), ever heard about wet-nursing, consent from the spouse, and milk preference in a serious medical condition. We first examined the association between the dependent and independent variables at the bivariate level, then entered all the variables into the model for multivariate analysis. We presented mean differences and 95% confidence intervals to express the associations between the dependent variable (acceptability of the use of donated breast milk) and independent variables. The results obtained were presented in the form of Tables and graphs. All analyses were done in Stata statistical software version 14.

## Results

### Participant characteristics

A total of 244 pregnant women, 144 from Nsambya and 100 from Naguru hospitals, were enrolled. The respondents’ ranged in age from 17 to 49, with a mean of 30 (± 5.25) years, and the majority were in the age group of 25–44 years. Half of the respondents attained the secondary level of education (50%), followed by university (22.9%). Almost all respondents (96.3%) were married/ cohabiting, had a normal delivery, and 73.4% had 1–3 children. Most of the respondents were Christians (82%), and only 18.0% belonged to Islam. Close to half of the respondents were housewives (40.6%), followed by self-employed (39.3%) and salaried or waged (20.1%). In addition, the mean monthly family income of the respondents was 274.64$ (± 457.80$). Details of respondents’ sociodemographic characteristics are presented in Table 2.

**Table 2:** Sociodemographic characteristics of the respondents (*n* = 244)

Variables	Category	Frequency	Percent
Marital status	Single	8	3.3
	Married or cohabiting	235	96.3
	Divorced or separated	1	0.4
Education level	Primary	38	15.6
	Secondary	122	50
	Vocational/technical	28	11.5
	University	56	22.9
Mode of delivery of current child	Cesarean section	67	27.5
	Normal delivery	177	72.5
Occupation status	Housewife	99	40.6
	Self-employed	96	39.3
	Salaried or waged	49	20.1
Religion	Christianity	200	82
	Islam	44	18.0
Age group	15–24	32	13.1
	25–44	211	86.5
	45 and above	1	0.4
Income	≤ 84$	78	31.9
	112$ -139$	46	18.9
	153$ -278$	76	31.2
	334$ -417$	44	18.0
Parity	1–3 children	179	73.4
	4 or more children	65	26.6

According to Table 3, half of the women had ever been counselled on breastfeeding during antenatal. 67.6% of the women had visited the antenatal unit at least 1–3 times. The majority of the respondents (81.1%) had never visited the neonatal intensive care unit. only 32.8% had ever heard about donated breast milk, and 24.6% heard about donated breast milk banking. More than half of the respondents (76.2%) had heard about wet nursing, but only 3.3% had practised it. In addition, 52.5% of the respondents preferred the use of donated breast milk if they were unable to breastfeed as compared to other feeds. The majority preferred relatives (50.4%) as donors followed by the human milk bank (38.9%). Most of the respondents (82.8%) stated that they would require consent from their spouse before feeding their babies on donated breast milk.

**Table 3:** Summary statistics for independent parameters (*n* = 244)

Variables	Category	Frequency	Percent
Ever heard about DBM	Yes	80	32.8
	No	164	67.2
Times visited the ANC	1–3 times	165	67.6
	4 or more times	79	32.4
Ever been counselled on breastfeeding during ANC visits	Yes	122	50
	No	122	50
Ever visited/been admitted to the NICU	Ever	46	18.9
	Never	198	81.1
Ever heard about DBMB	Yes	60	24.6
	No	184	75.4
Ever heard about wet-nursing	Yes	186	76.2
	No	58	23.8
Ever breastfed another person’s child	Yes	8	3.3
	No	236	96.7
Need spouse consent to use DBM	Yes	202	82.8
	No	42	17.2
Preferred infant feed in a serious medical condition	Donated breast milk	128	52.5
	Other feeds	116	47.5
Preferred donor	Relative	123	50.4
	Friend	5	2.1
	Human milk bank	95	38.9
	None	21	8.6

### Acceptability of the use of donated breast milk among pregnant women

**Fig. 1:**
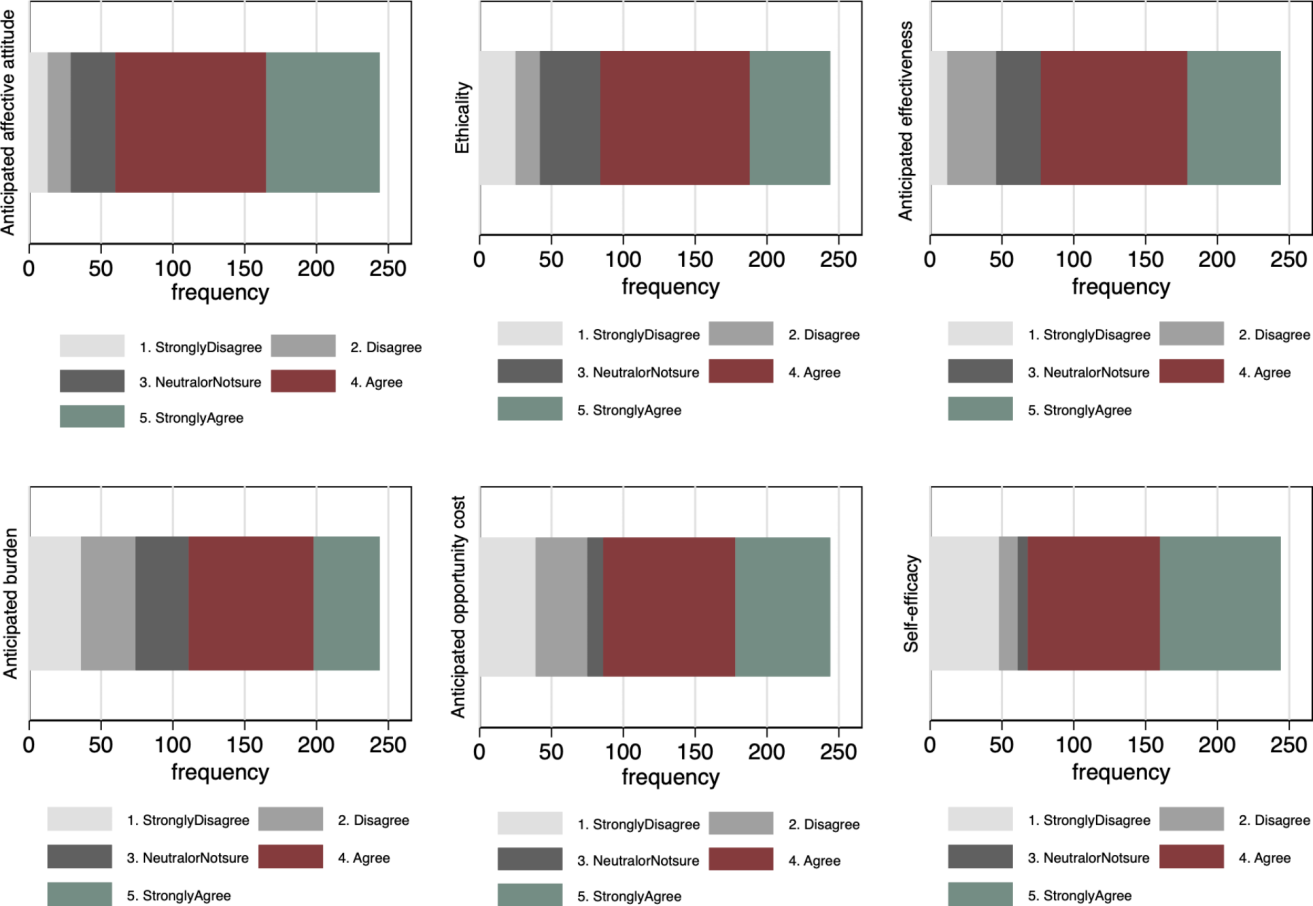
Distribution of responses on the acceptability of DBM across TFA constructs (*n* = 244)

Overall, 150/244 (61.5%) of the women agreed that they would accept feeding their babies on donated breast milk. The total acceptability score ranged from 6 to 30, with a mean of 21.58 (± 4.35). The distribution of responses across the six constructs used to measure the acceptability of donated breast milk is presented in Fig. 1. Regarding the anticipated affective attitude, 32.4% (*n* = 79) of the respondents strongly agreed that donated breast milk provides the best alternative source of food in the absence of the biological mother’s milk, whereas 5.3% (*n* = 13) strongly disagreed. 23% (*n* = 56) of the respondents strongly believed that it’s ethically acceptable to use donated breast milk for infant feeding, and 10.2% (*n* = 25) strongly disagreed. In terms of anticipated effectiveness, 26.7% (*n* = 65) of the women strongly agreed that donated breast milk would be effective, 41.9% (*n* = 102) agreed, 13.9% (*n* = 34) disagreed, and only 4.9% (*n* = 12) strongly disagreed. 18.8% (*n* = 46) of the respondents strongly agreed, and 35.7% (*n* = 87) agreed that using donated breast milk would be a burden in terms of expenses incurred, time and complexity of the process. However, 15.6% (*n* = 38) of them disagreed and only 14.7% (*n* = 36) strongly disagreed. 27.1% (*n* = 66) of the women strongly agreed, and 37.7% (*n* = 92) agreed that they would give up the convenience of using other baby feeds for donated breast milk. In terms of self-efficacy, 34.4% (*n* = 84) of respondents strongly believed that they would use donated breast milk, 37.7% (*n* = 92) agreed, 5.3% (*n* = 13) disagreed, and 19.7% (*n* = 48) strongly disagreed, while the rest were not sure.

### Factors associated with acceptability of the use of donated breast milk

Table 4 shows the results of bivariable and multivariable regression analysis conducted to identify factors associated with the acceptability of donated breast milk. Multivariable regression showed that the mother’s religion, having heard of donated breast milk banking before, higher education, and the presence of a serious medical condition were factors associated with acceptability. The average acceptability score of donated breast milk was 1.33 and 0.47 for women who had vocational/technical and university education, respectively, higher than that of women who had primary education (adjusted mean difference, vocational/technical versus primary level, 1.33 (95% CI 0.64 to 2.02) and university versus primary level, 0.47 (95% CI 0.001 to 0.93). The average acceptability score of donated breast milk was 1.24 higher among women who were Muslims compared to those who were Christians (adjusted mean difference,1.24; 95% CI 0.77 to 1.70). The average acceptability score of donated breast milk in the presence of a serious medical condition was 3.96 higher than that of other feeds (adjusted mean difference, 3.96; 95% CI 3.28 to 4.64). Women who had ever heard of donated breast milk banking scored 0.62 average acceptability higher than women who had never heard of donated breast milk banking (adjusted mean difference,0.62; 95% CI 0.18 to 1.06).

**Table 4:** Factors associated with the acceptability of the use of DBM among pregnant women

		Bivariable	Multivariable
	Mean acceptability score	Unadjusted mean difference (95% Cl)	Adjusted mean difference (95% Cl)
**Health facility**			
Nsambya Hospital	22.17 ± 4.18	0	0
Naguru Hospital	20.74 ± 4.48	-1.43 [-2.54, -0.32]	-1.13 [-1.54, -0.72]
**Age group**			
15–24	20.03 ± 5.02	0	0
25–44	21.82 ± 4.22	1.79 [-0.60, 4.18]	0.60 [-0.81, 2.01]
45 and above	21	0.97 [-2.18, 4.12]	0.28 [-3.07, 3.63]
**Education level**			
Primary	21.16 ± 4.23	0	0
Secondary	20.86 ± 4.24	-0.30 [-1.48, 0.89]	-0.60 [-1.31, 0.11]
Vocational/technical	23.21 ± 2.56	2.06 [0.75, 3.36]	1.33 [0.64, 2.02]
University	22.63 ± 5.01	1.47 [-1.07, 4.01]	0.47 [0.001, 0.93]
**Occupation status**			
Housewife	21.69 ± 4.45	0	0
Self-employed	21.24 ± 4.08	-0.45 [-2.45, 1.55]	-0.85 [-2.67, 0.97]
Salaried/waged	22.04 ± 4.71	0.35 [-1.70, 2.41]	-0.54 [-4.02, 2.93]
**Religion**			
Christianity	21.39 ± 4.45	0	0
Islam	22.46 ± 3.81	1.07 [0.93, 1.20]	1.24 [0.77,1.70]
**Times visited ANC**			
1–3 times	21.56 ± 4.32	0	0
≥ 4 times	21.63 ± 4.46	0.08 [-1.09,1.24]	-0.08 [-0.17, 0.02]
**Ever been counseled at ANC**			
Yes	22.21 ± 4.36	1.26 [0.45, 2.08]	0.74 [-0.68, 2.15]
No	20.95 ± 4.28	0	0
**Ever visited/been at NICU**			
Ever	22.83 ± 3.36	1.53 [1.19, 1.88]	0.23 [-0.06, 0.52]
Never	21.29 ± 4.51	0	0
**Ever heard about DBMB**			
Yes	22.90 ± 3.61	1.75 [0.44, 3.05]	0.62 [0.18, 1.06]
No	21.15 ± 4.49	0	0
**Ever heard about wet-nursing**			
Yes	22.01 ± 4.02	1.80 [0.09, 3.52]	1.30 [-0.34, 2.94]
No	20.21 ± 5.08	0	0
**Require consent from spouse**			
Yes	21.86 ± 3.93	1.60 [-1.87, 5.06]	1.61[-1.30, 4.52]
No	20.26 ± 5.86	0	0
**Presence of a serious medical condition**			
Other feeds	19.35 ± 4.84	0	0
Donated breast milk	23.61 ± 2.52	4.27 [5.43, 3.10]	3.96 [3.28, 4.64]

## Discussion

This study sought to assess the acceptability and factors associated with the use of donated breast milk among pregnant women. The study revealed that most (61.5%) women would accept giving their babies donated breast milk. This finding is consistent with a study conducted in southeast Nigeria among pregnant and breastfeeding mothers, where 60% of the mothers were willing to use donated breast milk [4]. Similarly, Kimani-Murage et al. (2019) reported that 59% of respondents at the individual level in Kenya found using donated breast milk acceptable [13]. Furthermore, a study conducted in Bhopal, India, reported that 85.4% of the women surveyed were willing to accept donated breast milk [35]. Contrary to these findings, a mixed-methods study in eastern Ethiopia conducted among mothers revealed that only 15.2% of respondents were willing to feed their babies donated breast milk [14]. A cross-sectional study conducted at the University of Benin Teaching Hospital, Nigeria, reported that 84.8% of the mothers would not give their babies donated breast milk [15]. In China, 38.3% of the 1078 postpartum mothers surveyed accepted donated breast milk [36]. The high acceptability in this study could be because one of the study sites had ongoing human milk banking activities, which might have exposed women to the practice and idea of using and banking breast milk before the study. Also, the experience and counselling on breastfeeding received by mothers during antenatal care might have contributed to the acceptance of donated breast milk. These findings agree with a mixed-methods study conducted in eastern Ethiopia, which found that mothers who had ever been counselled about breastfeeding were more likely to accept donated breast milk [14]. These findings suggest that integrating donated breast milk into breastfeeding promotion campaigns and health education at antenatal clinics would make it acceptable.

In this study, women who had heard of donated breast milk banking had higher acceptability than those who had never heard of it. Similarly, a mixed-method study in eastern Ethiopia reported that mothers who had heard of donated breast milk banking were 5.8 times more likely to accept donated milk than those who had never heard of it before [14]. Correspondingly, a cross-sectional multi-centre study in southeast Nigeria reported that knowledge of donated milk was a significant predictor of accepting to receive donated breast milk [4]. A study in Wuhan, China, reported having prior knowledge of human milk banking as a factor that positively predicts acceptance of donated breast milk [36]. These findings emphasize the importance of creating public sensitization on human milk banking. Furthermore, in our study, women with a high level of education had high acceptability of donated breast milk. These findings are similar to those of a study conducted in New York, which investigated the attitudes of postpartum women towards donor milk, and established that 64% of the respondents with education beyond high school accepted that donated milk was more beneficial to babies than those with high school education or less [7]. Studies in Kenya, southeast Nigeria and China demonstrated that educated mothers tend to have knowledge of donated breast milk and are more likely to involve in human milk banking [4, 13, 37]. Contrary to these findings, another study conducted in Turkey among married women reported that women with education status less than secondary school (83.6%) were more likely to accept breast milk sharing than those with education beyond secondary level or higher [38]. These results could be due to the exposure and the ability to process and comprehend information that comes with higher education. This finding suggests a need to design targeted education programs about human milk banking and focus on women with lower education.

Other factors significantly associated with the acceptability of using donated breast milk found in this study included; the mother’s religion and the presence of a severe medical condition. Women preferred using donated breast milk to other feeds when breastfeeding is impossible. These findings are similar to a study conducted in south-east Nigeria, where mothers agreed to use donated breast milk than infant formula in case of a serious medical condition [4]. Also, a cross-sectional study conducted in Nairobi, Kenya, reported that at the personal level, 59% of mothers would accept their children to feed on donated milk from a human milk bank in case of any condition limiting breastfeeding [13]. Likewise, an online survey on human milk sharing in the United States reported that respondents fed their babies donated breast milk because of a medical condition [39]. Correspondingly, 47.5% of mothers in Izmir, Turkey, would use the donated breast milk if they could not breastfeed [23]. However, only 26.7% of mothers were willing to use donated breast milk without any condition hindering breastfeeding [23]. The preference for donated milk in a severe medical condition could be due to the knowledge of mothers on the health and economic benefits of breast milk. Kair and Flaherman (2017) reported in their study that mothers choose donated breast milk over formula for health reasons [40]. Donated breast milk is recommended as the ideal food for a baby whenever a mother cannot breastfeed, especially among those with a medical condition [3, 41]. On the other hand, our findings indicating a positive relationship between being a Muslim and the acceptability of donated milk differs from a cross-sectional study conducted in Turkey [23]. The study reported that 37.2% of mothers expressed religion as a reason for not accepting donated milk use and banking [23]. Similarly, in a study by Gürol et al., 36.3% of the women surveyed did not accept donated breast milk because of religion [42]. Donated breast milk is considered unacceptable among Muslim communities because of the concern about marriage between milk siblings [42–45]. We don’t know why there was high acceptability for donated milk among the Muslim respondents in the current study.

### Limitations

The study findings were limited to the urban and hospital context. The level of acceptability of using donated breast milk might not be the same in community and rural areas.

## Conclusions

The acceptability of using donated breast milk for infant feeding was high among pregnant women. Thus, the Ministry of Health and its partners should establish human milk banks in key health facilities to increase the availability and accessibility of donated breast milk for mothers in need, especially those with serious medical conditions. Public sensitization and education campaigns are indispensable for the acceptability of donated milk. These programs should be designed to include women with lower education levels. Further research is needed to understand the relationship between religion and the acceptability of donated breast milk in Uganda.

## Electronic supplementary material

Below is the link to the electronic supplementary material.

**Additional Figure 1** - Normal probability plots

**Additional Figure 2** - Fitted values VS. residuals

## Data Availability

The datasets used and/or analyzed during the current study are available from the corresponding author upon reasonable request.

## Abbreviations

DBM: Donated Breast Milk
DBMB: Donated Breast Milk Banking
NICU: Neonatal Intensive Care Unit
TFA: Theoretical Framework of Acceptability
WHO: World Health Organization
