# Supplementary figures and images for: Acceptability of donated breast milk among pregnant women in selected hospitals in central Uganda: a cross-sectional study
Source: Int Breastfeed J. 2023 Jun 16;18:32. doi: 10.1186/s13006-023-00569-x (PMC10276413; doi:10.1186/s13006-023-00569-x)

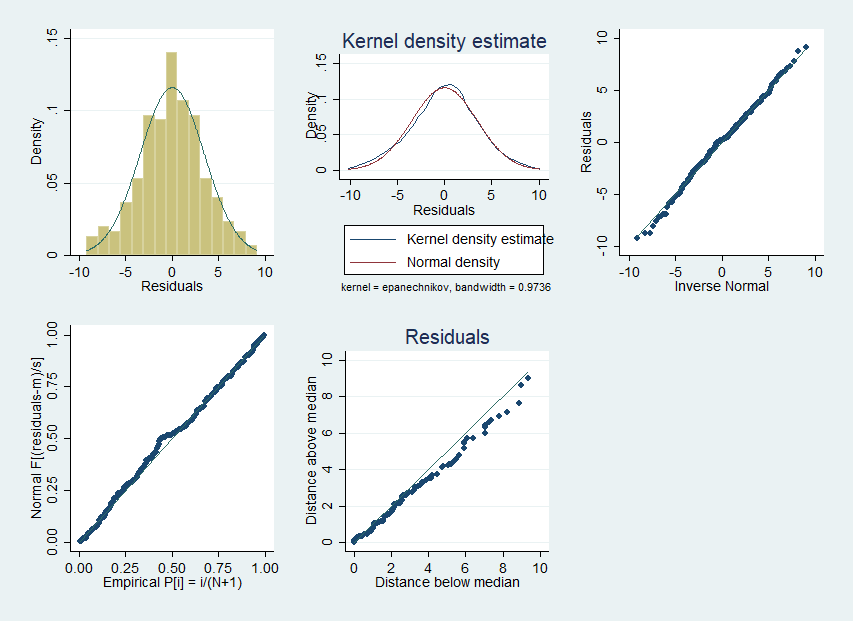

Supplement: Supplementary file 1 — Additional Figure 1 - Normal probability plots [file 13006_2023_569_MOESM1_ESM.png]

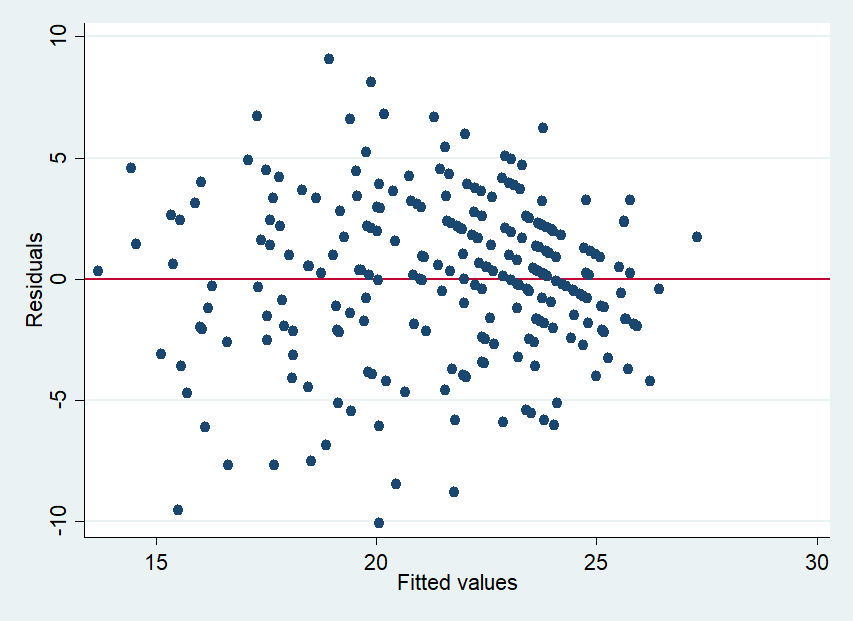

Supplement: Supplementary file 2 — Additional Figure 2 - Fitted values VS. residuals [file 13006_2023_569_MOESM2_ESM.png]
